# Supplementary figures and images for: Wheat Sensitivity and Functional Dyspepsia: A Pilot, Double-Blind, Randomized, Placebo-Controlled Dietary Crossover Trial with Novel Challenge Protocol
Source: Nutrients. 2020 Jun 30;12(7):1947. doi: 10.3390/nu12071947 (PMC7400003; doi:10.3390/nu12071947)

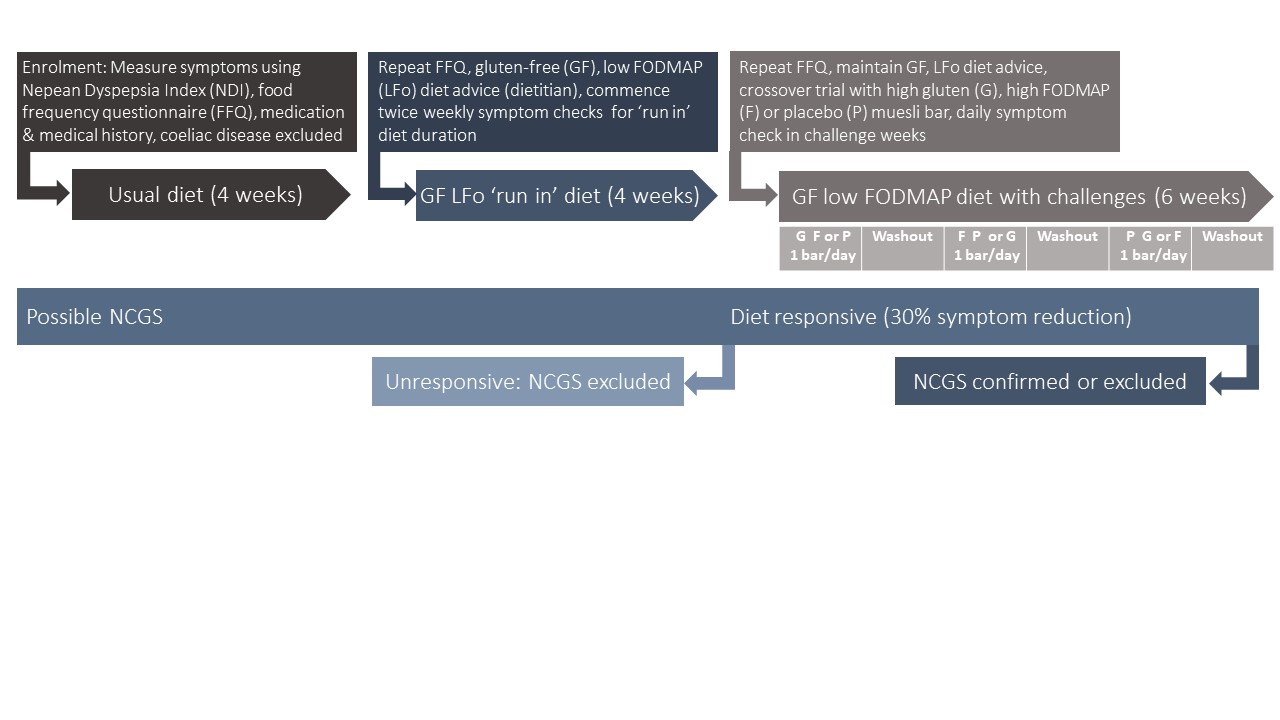

Supplement: Supplementary file 1 [file nutrients-12-01947-s001.zip › Figures_NCWS_MP/Slide1.JPG]

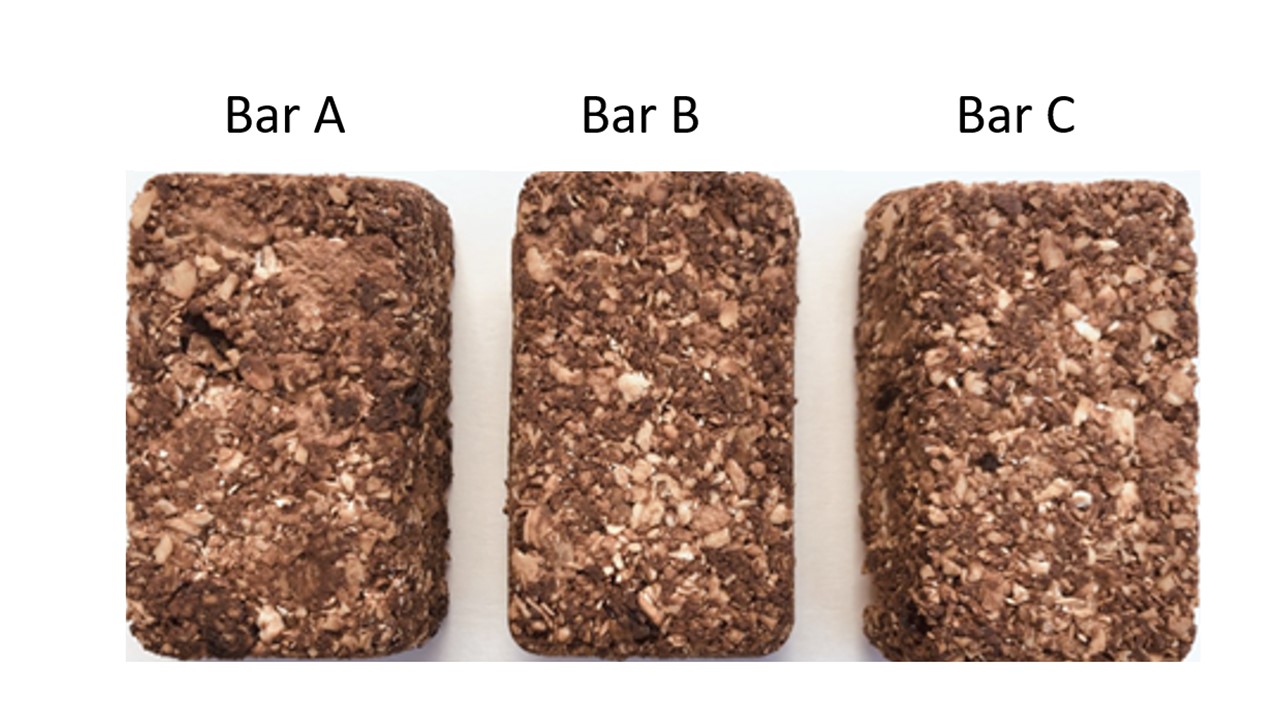

Supplement: Supplementary file 1 [file nutrients-12-01947-s001.zip › Figures_NCWS_MP/Slide2.JPG]

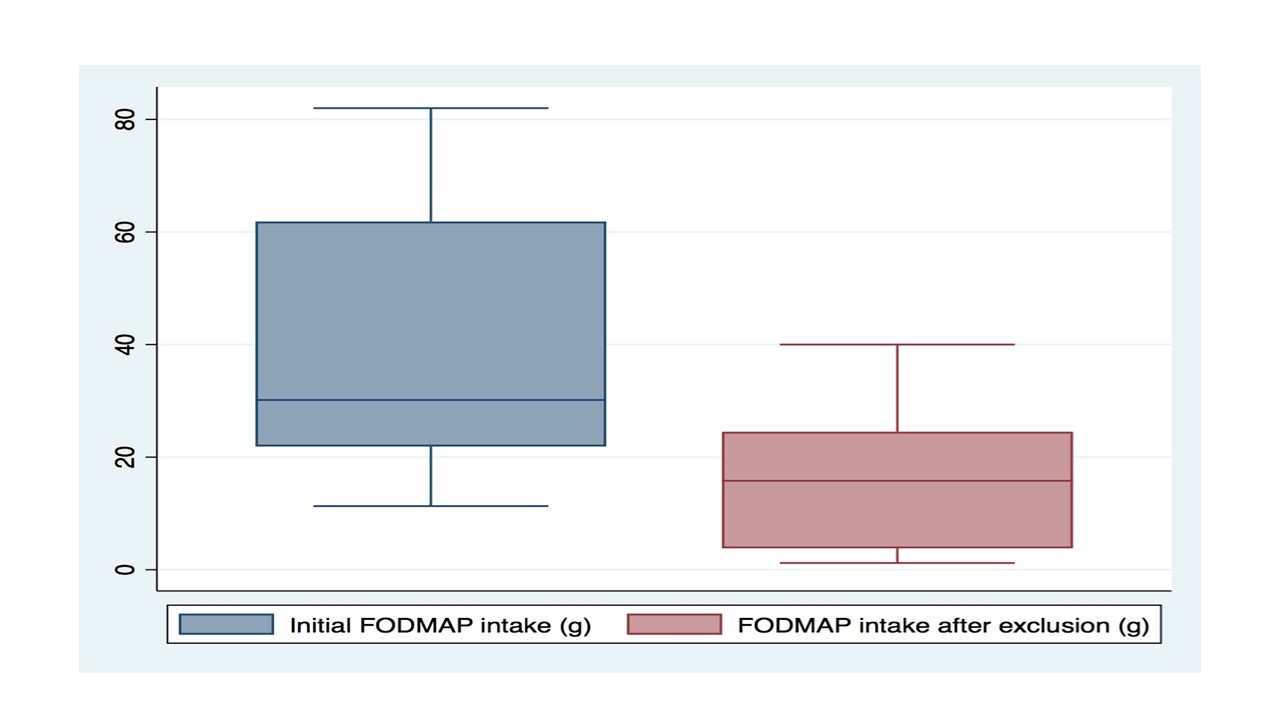

Supplement: Supplementary file 1 [file nutrients-12-01947-s001.zip › Figures_NCWS_MP/Slide3.JPG]

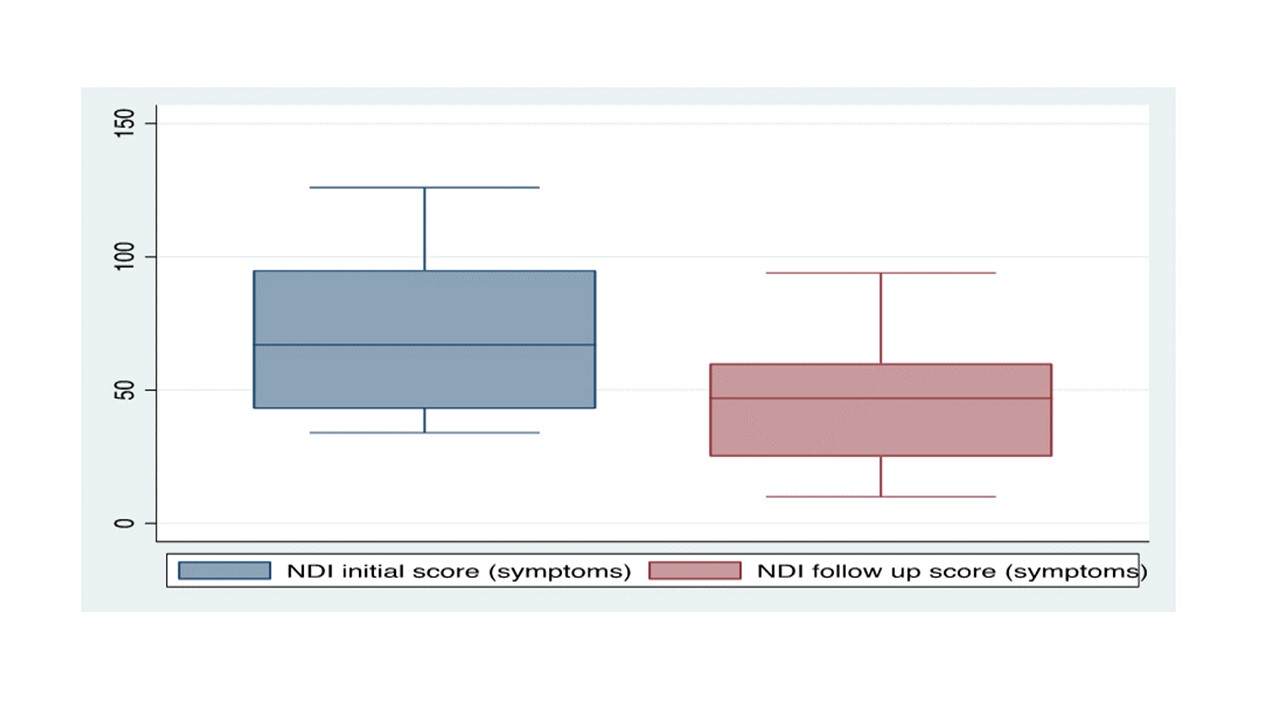

Supplement: Supplementary file 1 [file nutrients-12-01947-s001.zip › Figures_NCWS_MP/Slide4.JPG]

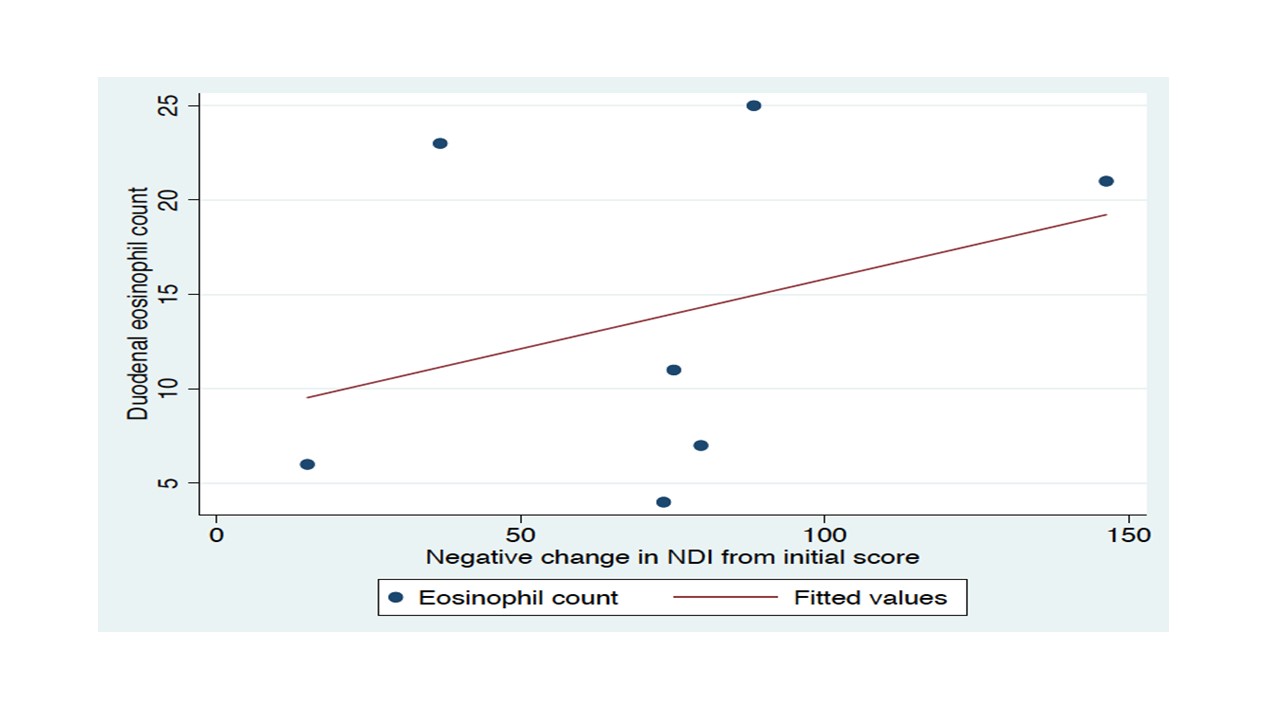

Supplement: Supplementary file 1 [file nutrients-12-01947-s001.zip › Figures_NCWS_MP/Slide5.JPG]

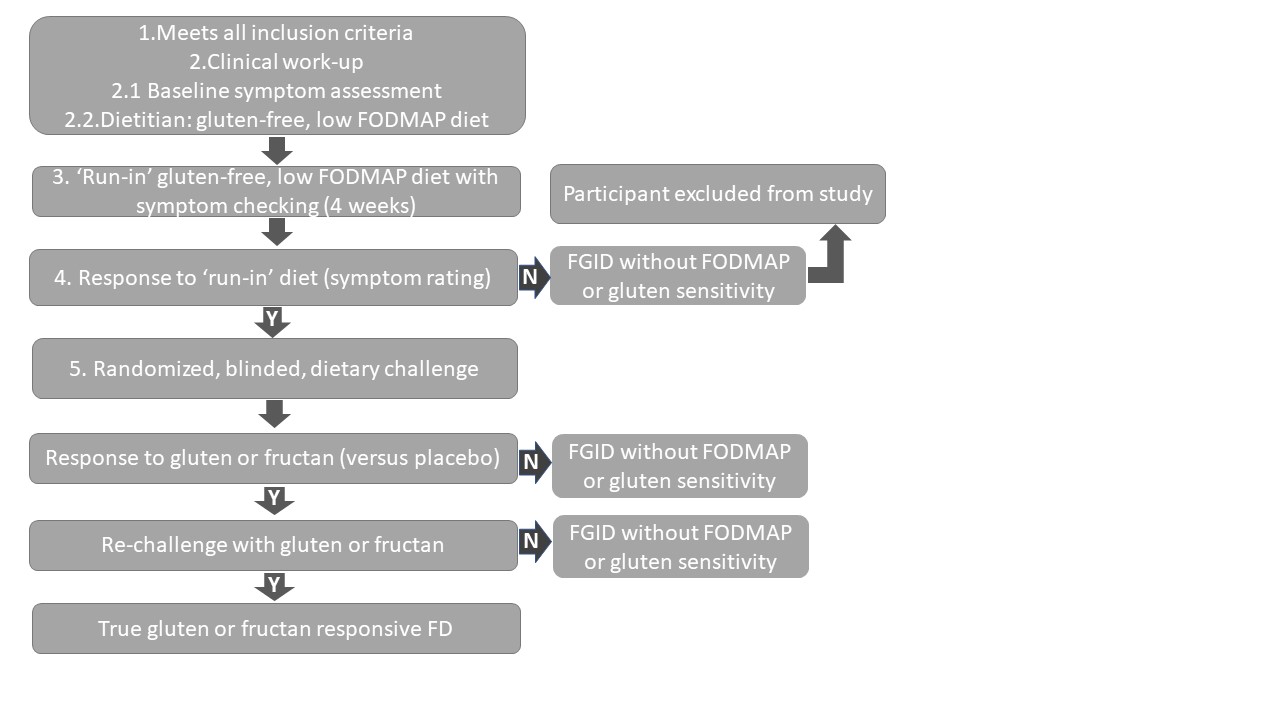

Supplement: Supplementary file 1 [file nutrients-12-01947-s001.zip › Figures_NCWS_MP/Slide6.JPG]

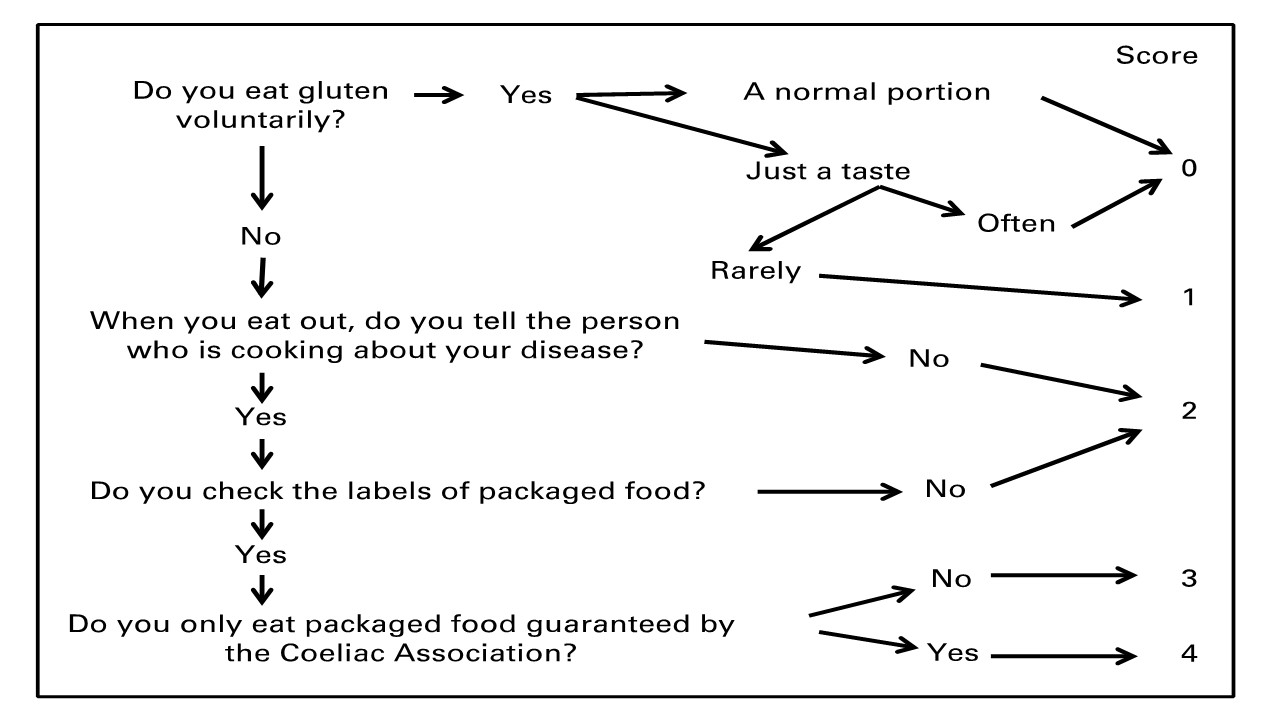

Supplement: Supplementary file 1 [file nutrients-12-01947-s001.zip › Figures_NCWS_MP/Slide7.JPG]
